# Characterizing two surface states and their role in the photoinduced oxygen evolution reaction on hematite *via* photocurrent kinetics

**DOI:** 10.1039/d5cp04300j

**Published:** 2026-03-12

**Authors:** Yuke Yang, Felix Zerres, Soma Salamon, Georg Bendt, Stephan Schulz, Heiko Wende, Yujin Tong, R. Kramer Campen

**Affiliations:** a Faculty of Physics, University of Duisburg-Essen, Lotharstr. 1 47057 Duisburg Germany richard.campen@uni-due.de 0203/379 4690; b Faculty of Chemistry, University of Duisburg-Essen, Universitätsstr. 7 45141 Essen Germany; c Center for Nanointegration Duisburg-Essen (CENIDE), University of Duisburg-Essen, Lotharstr. 1 47057 Duisburg Germany

## Abstract

Hematite (α-Fe_2_O_3_) is a promising photoanode for solar water splitting, whose efficiency is limited by rapid charge recombination, sluggish hole transport and slow oxygen evolution reaction kinetics. Understanding which of these factors actually leads to inefficiency, *i.e.* non-unitary photon conversion, is challenging. Here we show, for a model hematite photoanode, that analysis of wavelength-dependent (405–645 nm) photocurrent kinetics as a function of bias (0.9–1.65 V *vs.* RHE) reveals two surface states. The observed bias dependence and relative size of the charge transfer resistances and capacitances associated with each state are most easily rationalized if our α-Fe_2_O_3_(0001) anode is characterized by a mixed Fe/O termination that results in populations of monodentate and bidentate coordinated surface oxygens. Bidentate coordinated surface O(H) are the active site for the photoinduced OER but populations of monodentate surface OH change in response to applied bias/illumination in a manner that controls surface charge. At potentials where OER occurs in the dark, both sites are catalytically active.

## Abstract 2

Bias-dependent, λ-resolved photocurrent kinetics and interfacial capacitor analysis separate bulk and surface processes on hematite, revealing monodentate and bidentate OH with distinct kinetics and showing the OER proceeds *via* bidentate OH.

## Graphical abstract

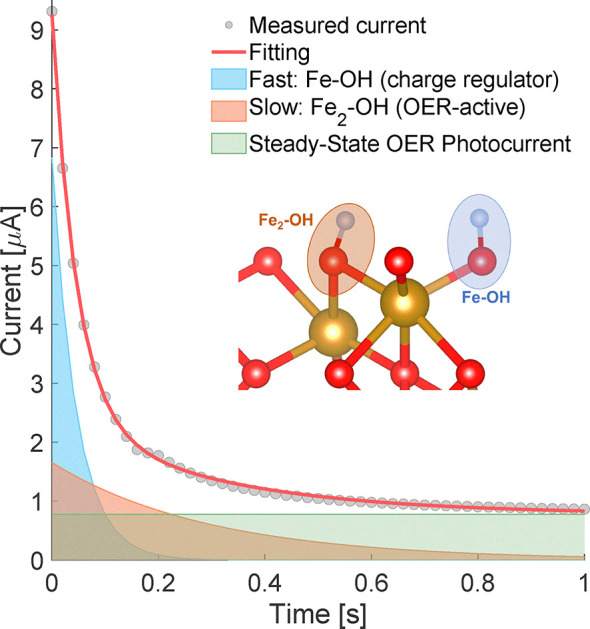

## 1 Introduction

Hematite (α-Fe_2_O_3_) is a promising photoanode for solar water splitting due to its bandgap (≈ 2.1 eV), valence band energy alignment, stability in alkaline solution and natural abundance.^1–4^ However, its solar-to-hydrogen (STH) efficiency is limited by rapid bulk charge recombination,^5^ short hole diffusion length (2–4 nm)^3,6,7^ and sluggish surface oxygen evolution reaction (OER) kinetics.^8^ While n-doping and nanostructuring have partly overcome these challenges, current state-of-the-art hematite anodes yield photocurrent densities of 4–5 mA cm^−2^ at 1.23 V *vs.* RHE, far below the theoretical maximum of 12.6 mA cm^−2^ at AM 1.5G illumination.^7,9–13^ From a microscopic perspective such inefficiency suggests that for some fraction of absorbed photons electron/hole recombination outcompetes the use of holes to catalyze the oxygen evolution reaction (OER) and extraction of electrons from the device. Recent work by Graves and coworkers has highlighted that hematite's incident photon-to-current efficiency (IPCE) drops off sharply at wavelengths longer than 500 nm.^14,15^

At ≈ 420 nm hematite absorption reaches a maximum and is dominated by a ligand-to-metal charge transition (LMCT) resulting in an O centered hole. At both longer and shorter wavelengths, particularly at wavelengths longer than 500 nm, absorption is increasingly dominated by ligand field (d–d) spin flip transitions that produce an Fe centered hole.^16,17^ As illustrated by Hayes *et al.* computing the absorption spectrum accounting only for LMCT transitions appears to quantitatively reproduce the IPCE spectrum.^17^ While suggestive, this correlation does not offer mechanistic insight. Prior studies have concluded that the microscopic origin of this “non-unitary photon conversion” in hematite is ultrafast trapping, *i.e.* small polaron formation,^18^ electron/hole recombination^5^ or both. It is, however, not clear how these, or other possible elementary processes, produce the observed difference between absorption and IPCE spectra.

Since photocurrent is the fundamental metric of interest we follow prior workers by partitioning the signal into components,^14–19^

1 *J*_photo_ = *J*_abs_·*ξ*_pg_·*η*_cs_·*η*_ct_

in which *J*_photo_ is the measured photocurrent density, *J*_abs_ is the maximum photocurrent density under AM 1.5G illumination assuming unitary photon conversion, *ξ*_pg_ the photogeneration efficiency, *η*_cs_ is charge separation efficiency and *η*_ct_ charge transfer efficiency.

Eqn (1) clarifies that photocurrent density in hematite is influenced by three factors: 1) Effective photogeneration yield (*J*_abs_·*ξ*_pg_): the fraction of absorbed photons generating mobile carriers, limited by wavelength-dependent absorption and ultrafast trapping. Both *J*_abs_ and *ξ*_pg_ are wavelength-dependent but potential-independent: both depend on the photophysics of bulk hematite. 2) Charge separation efficiency (*η*_cs_): determined by carrier mobility, recombination, external bias and illumination intensity (*i.e.* photovoltage). *η*_cs_ is expected to depend on both wavelength and bias. 3) Charge transfer efficiency (*η*_ct_): the competition between catalytic turnover (*e.g.* population of Fe–OOH intermediates) and surface-state recombination. *η*_ct_ is expected to depend both on wavelength and applied bias. Resolving the relative contributions of these factors to hematite photocurrent is important because individual modification strategies do not improve all simultaneously.^20^ To gain this insight we proceed here by examining the wavelength dependence of these three contributors to the measured IPCE.

Prior experimental efforts to disentangle the contributions to eqn (1) largely consider either steady-state macroscopic (*e.g.* how measured currents or photovoltages depend on applied bias or illumination) or steady-state microscopic observables (*e.g.* optical/X-ray spectroscopies or scanning probe microscopies).^20,21^ Connecting these two observable types, *e.g.* understanding the microscopic mechanism underlying a particular charge transfer resistance identified in electrochemical impedance spectroscopy (EIS) under illumination, is challenging.

Describing electron transfer across the hematite/water interface requires describing the journey of the electron/hole pair from their creation within bulk hematite, loss due to ultrafast bulk recombination, transport within bulk hematite (for the electron to the back contact, for the hole to the hematite/water interface) and any surface-mediated recombination or catalytic chemistry. Much prior work has shown that these elementary processes differ both in their characteristic timescales and in their dependence on charge carrier energy. Non-steady-state photoelectrochemical device level characterization, *e.g.* intensity modulated photocurrent or photovoltage spectroscopies,^22–24^ offers the possibility of separating charge relaxation processes that occur on timescales from milliseconds to seconds and thus offers a partial way to overcome this problem. However, virtually all work along these lines to this point has either employed chopped simulated solar or single wavelength monochromatic illumination. While important, such approaches make it difficult to understand the dependence of the elementary processes they sample on charge carrier energy.

In this study, we perform transient photocurrent analysis, *i.e.* photochronoamperometry, following narrow-band monochromatic excitation covering 405–645 nm. The resulting deconvoluted *J*_abs_·*ξ*_pg_, *η*_cs_ and *η*_ct_ are most simply understood if our α-Fe_2_O_3_ surface is characterized by a mixed Fe/O termination resulting in both monodentate and bidentate coordinated surface hydroxyls. The relative size and bias dependence of resistances and capacitances associated with each population suggest bidentate surface OH are the active site for the OER, but monodentate surface OH population changes as a function of applied bias and illumination as it compensates changes in surface polarization.

## 2 Experiments

### 2.1 Preparation of hematite sample

Following prior workers we fabricated hematite photoanodes, underlain by thin platinum (Pt) film on a c-cut sapphire substrate using pulsed laser deposition (PLD).^10^ The substrates (Alineason Materials Technology GmbH; dimensions: 10 × 10 × 0.5 mm^3^) were ultrasonicated in acetone, ethanol, and Milli-Q water for 15 minutes each prior to deposition. PLD was conducted in a high-vacuum chamber (base pressure: 10^−8^ mbar). A 60 nm Pt interlayer was first deposited using a KrF excimer laser (COMPexPro 102, Coherent LaserSystems GmbH & Co. KG; wavelength *λ* = 248 nm, pulse energy = 200 mJ, repetition rate = 10 Hz) ablating a 99.99% pure Pt target (Alineason Materials Technology GmbH). Pt deposition was conducted with the sapphire substrate at 500 °C under dynamic pressure (10^−7^–10^−6^ mbar). A 30 nm hematite (α-Fe_2_O_3_) layer was subsequently deposited under identical laser conditions using a 99.99% pure α-Fe_2_O_3_ target (Alineason Materials Technology GmbH) with the Pt covered sapphire at 700 °C. The deposition rate was calibrated *in situ* using a quartz crystal microbalance positioned at the substrate location.

Post-deposition annealing was performed in a tube furnace (Carbolite CTF 12/75/700) in ambient air by (1) ramping from room temperature to 550 °C (∼2 °C s^−1^ heating rate), (2) holding the sample at 550 °C for two hours, (3) ramping to 800 °C with the same rate and holding for 20 minutes and (4) cooling in the furnace to 520 °C followed by air cooling.

### 2.2 Anode characterization

Post-annealing Raman spectra show only peaks characteristic of hematite: features characteristic of other Fe-oxides or Pt-oxides are absent (see Fig. S1). The crystal structure of the annealed sample was analyzed by X-ray diffraction (XRD). Reflections characteristic of Pt, α-Al_2_O_3_ and α-Fe_2_O_3_ are clearly observed along with trace contributions from Pt-oxides (see Fig. S2). The crystallographic structure and surface morphology of the post-annealing sample were further characterized by scanning electron microscopy. As shown in Fig. S3 these micrographs show our sample creation procedure produces hexagonal crystallized hematite grains with the (0001) facet predominantly exposed.

### 2.3 (Photo)electrochemistry

Cyclic voltammetry (CV) and chronoamperometry (CA) were performed in a custom PTFE (Teflon) three-electrode cell (see Fig. S4 for cell details) using a BioLogic SP200 potentiostat. A Pt wire and a Ag/AgCl electrode (BioLogic, RE-1BP) served as the counter and reference electrodes, respectively. The electrolyte consisted of 0.1 M NaOH (≥99.0% purity, Acros Organics) dissolved in ultrapure deionized water (18.18 MW cm, Stakpure OmniaPure UV), yielding a pH of 12.8. A peristaltic pump (Ismatec ISM596D) continuously circulated the electrolyte through the cell at a flow rate of 10 µL s^−1^.

Optical excitation was provided by a supercontinuum laser source (Leukos Rock 400 4, repetition rate: 60 MHz) with a spectral range of 400–2400 nm. A tunable filter (Leukos Bebop) was employed to narrow the output to a Gaussian spectrum with a full width at half maximum of 10 nm. The resulting narrow band output was scanned in 20 nm increments from 405 to 645 nm. Each illumination condition is hereafter referred to by its center wavelength. The collimated output of the laser was spatially filtered to ensure the diameter remained smaller than the exposed hematite working electrode. Pulsed illumination was generated by blocking the laser with an optical shutter (5-second on/off cycles), synchronized to initiate approximately 80 seconds after the start of CA measurements. CA measurements were performed at potentials ranging from 0.9 V to 1.6 V (*vs.* RHE) in 0.1 V increments. The sampling interval for all measurements was 20 ms. To maintain consistent sample conditions, a five-cycle CV scan (0.57–1.62 V *vs.* RHE, scan rate: 200 mV s^−1^) was conducted between successive CA steps. To enable direct comparison of photocurrents across all wavelengths, the incident photon flux density was set to 9.4 × 10^16^ cm^−2^ s^−1^ for all CA measurements. In addition, CA measurements were repeated in 0.1 M NaOH containing 1 mM H_2_O_2_ under otherwise identical conditions.

All photocurrent decay profiles were processed by averaging three consecutive light pulses and applying dark-current correction *via* linear baseline fitting. The resulting data were then analyzed using the nonlinear least-squares solver lsqnonlin (Optimization Toolbox, MATLAB R2023b). A detailed description of the data-processing workflow, including all intermediate steps and a flow chart, is provided in the SI (Fig. S5).

## 3 Results and discussion

**Fig. 1:**
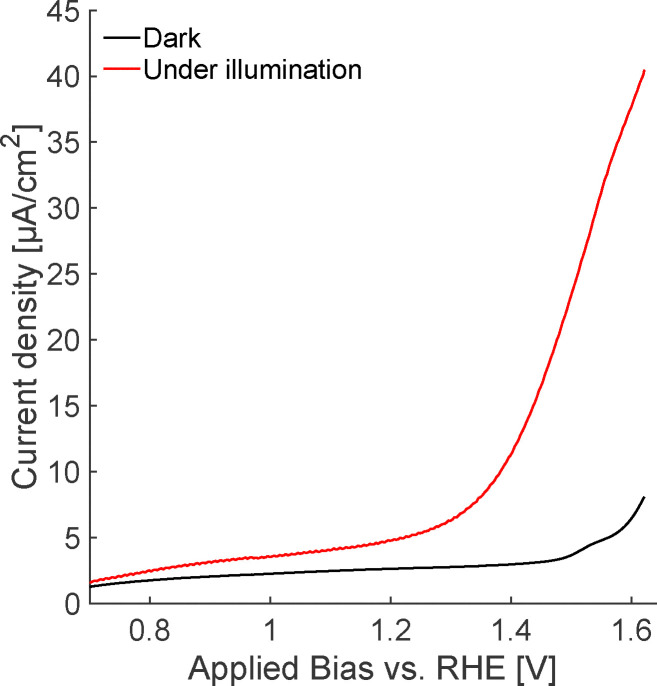
Positive CV sweep performed in the dark and under 405 nm supercontinuum laser illumination in 0.1 M NaOH solution. The laser power density was maintained at 46.1 mW cm^−2^, with photon density consistent with all chronoamperometry (CA) measurements.

High performance hematite photoanodes are nanostructured on 5–10 nm length scales.^9,25–27^ Such nanostructuring improves performance but makes understanding surface physical chemistry more complicated. Because our focus in this work is on understanding the mechanism of the OER on hematite, we here wish to create a sample that is relatively more crystalline than the benchmark nanostructured (thus likely with a lower photocurrent) but with a similar OER onset potential. Fig. 1 shows the anodic cyclic voltammetry (CV) sweep of hematite in 0.1 M NaOH under 405 nm illumination. A distinct photocurrent onset occurs at ∼1.3 V *vs.* RHE, consistent with prior work.^10,28^ The measured photocurrent density is ≈100 × lower than the state of the art under AM 1.5G illumination.^13^ While our hematite photoanode is 30 nm thick, SEM images (Fig. S3 in the SI) clearly indicate that our PLD + annealing procedure produces well-crystallized domains with characteristic sizes larger than 100 nm. Photoelectrochemical impedance spectroscopy results (see SI for data, Fig. S6 and S7, and methodological details) suggest that the dopant density (expected to be dominated by oxygen vacancies) is *N*_D_ ∼ 10^19^ cm^−3^ and that the interfacial capacitance increases smoothly with anodically increasing bias: consistent with an electrode with minimal Fermi level pinning and a space charge layer that fully decays within the hematite layer over the potential range of the experiment.^29,30^

**Fig. 2:**
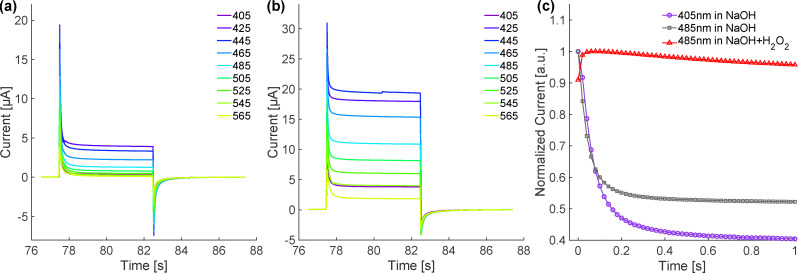
Photocurrent characteristics under CA conditions as a function of illumination wavelength and applied bias voltage. (a) Photocurrent measured at 1.3 V bias in a 0.1 M NaOH solution; (b) photocurrent measured at 1.5 V bias in a 0.1 M NaOH solution; (c) temporal decay profile of photocurrent during continuous laser illumination, the time zero of x-axis is calibrated to the current spike in (a) and (b). The addition of H_2_O_2_ strongly modifies the amplitudes, time constants (and sometimes the sign) of both exponential components suggesting both charge relaxation processes are interfacially mediated.

Given a sample appropriate to gain the mechanistic insight we seek, we next characterize the photocurrent kinetics. Fig. 2 shows representative pulsed photocurrent data as a function of laser wavelength and applied bias. At biases above the photocurrent onset the same qualitative response is observed at all wavelengths: a current overshoot on opening the shutter that relaxes to the, bias-dependent, steady-state photocurrent and an undershoot on closing the shutter before the current relaxes to zero. This response has been observed previously by multiple groups and rationalized by the following scenario.^21–25^ Turning on illumination results in an instantaneous hole current towards the interface. These holes are captured in surface states inducing an electron current towards the interface driving surface recombination and relaxation of the photocurrent towards the steady-state (electron current extracted from the device decreases). On turn-off of illumination the hole current is shut-off quasi-instantaneously and the remaining surface holes are consumed by a flow of electrons to the interface, *i.e.* a current that opposes the steady-state photocurrent. Physically the hematite surface and space charge region can be understood as a capacitor discharged by photo-generated surface holes trapped by formation of surface intermediates (*e.g.* Fe–OH) and recharged as electrons flow to the surface.

Comparison of Fig. 2(a) and (b) clarifies that the photogenerated current exhibits a distinct wavelength dependence, reaching its maximum value at 425–445 nm, and increases with increasing bias (at least from 1.3 to 1.5 V *vs.* RHE). Control experiments suggest that with the illumination intensity we employed photocurrent kinetics do not depend on incident fluence (Fig. S8 and S9 in the SI). Preliminary studies on our photoanode suggest that, following illumination, the current relaxes to its steady state on two distinct time scales, ≈50 ms and ≈2 s. Similar measurements in the presence of H_2_O_2_ (a hole scavenger^19^) also show two relaxation processes at similar timescales but with different amplitudes or signs (see Fig. 2(c)). Such H_2_O_2_–sensitive changes are consistent with a scenario in which both relaxation processes in the alkaline, H_2_O_2_-free solution, are mediated by surface processes, rather than by purely bulk transport or double-layer charging.

Given that both charge relaxation processes are a consequence of surface processes, we next quantify relaxation of both processes to the steady-state photocurrent, *i.e.* turn-on dynamics, using a dual-exponential decay model:2
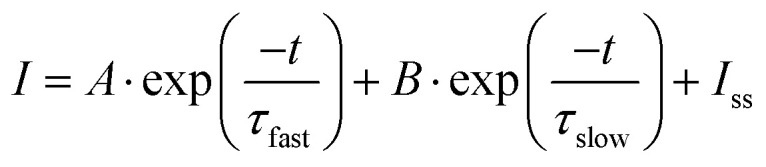
where *A* and *B* are the amplitudes [mA], *τ*_fast_ and *τ*_slow_ are the two time constants [s] and *I*_ss_ is the steady-state photocurrent [mA]. Note that formulated as in eqn (2) both decay processes reflect characteristic timescales for the charging of surface states ††This view of our data is equivalent to taking hole consumption to lead to a charging of an interfacial capacitor. From a mathematical perspective we could equivalently describe our signal as a loss from surface states *via* recombination: *i.e.*
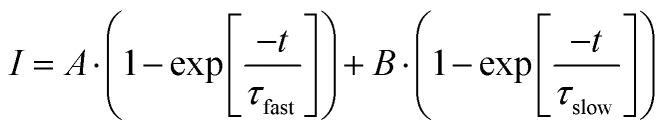
. Because in the surface state charging formulation makes the interpretation of *A* and *B* more straightforward (as discussed in the text) we here adapt this model..

**Fig. 3:**
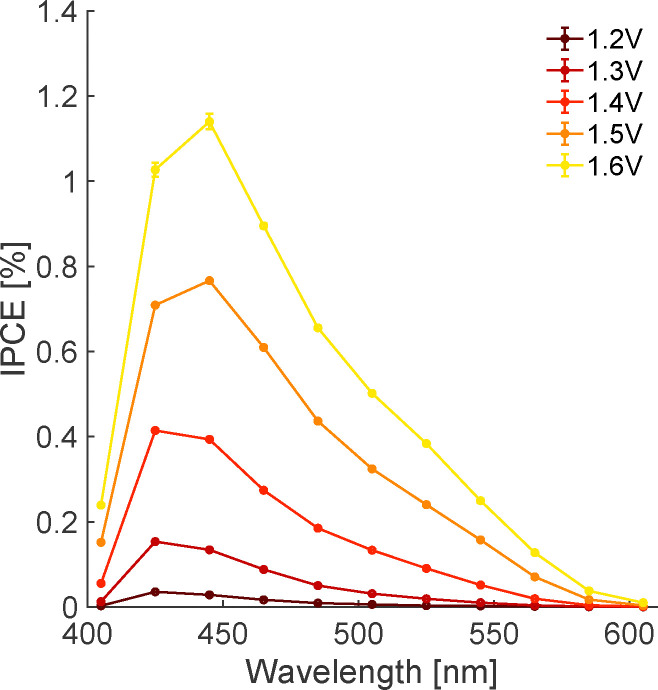
IPCE of hematite photoanodes under varying applied bias (1.2 to 1.6 V) in 0.1M electrolyte (pH = 12.8).

Fitting the photocurrent decay under illumination in this manner allows the extraction of *I*_ss_ and thus calculation of the wavelength-dependent incident photon-to-current efficiency,3
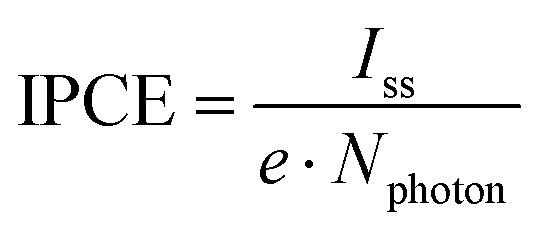
where *e* is the elementary charge and *N*_photon_ is the incident photon flux. As shown in Fig. 3, we find IPCE low near 405 nm, increasing to a maximum between 425 and 445 nm, decreasing more slowly with increasing wavelength to 600 nm and increasing with bias. The photocurrent onset near 600 nm corresponds to an optical bandgap of ∼2.0 eV for this sample, consistent with literature values (1.9–2.2 eV).^31–33^ Prior IPCE measurements in hematite photoanode devices have observed a similar response, but with a smaller decrease near 405 nm.^9,17,34–38^ To rationalize the relatively pronounced attenuation of the IPCE at 405 nm, we performed a thin-film optical calculation for the actual electrode architecture (electrolyte/hematite(30 nm)/Pt) using a coherent transfer-matrix method (see Fig. S12 and detailed description in the SI). The model yields the hematite-layer absorptance *A*_hem_(*λ*): the fraction of incident optical power dissipated within the 30 nm hematite film after accounting for multiple reflections and interference enabled by the Pt back contact. The results clarify that the IPCE attenuation at 405 nm can be understood as the combined consequence of (i) an intrinsically weaker optical transition strength of hematite around ∼400–420 nm,^17^ which lowers the number of photogenerated carriers available to contribute to *I*_ss_ and (ii) destructive interference with reflection from the Pt/hematite back contact, *i.e.* reduced *A*_hema_ (405 nm) in the air/hematite/Pt stack relative to the hematite layer under otherwise identical photon flux. Under our constant-photon-flux protocol, these two effects act in the same direction, directly suppressing *I*_ss_ (405 nm) and thus attenuating the IPCE at 405 nm. Prior IPCE reports were measured on devices that do not have a Pt back contact and generally are not performed with wavelength-independent photon flux.^9^

Given an overpotential for the OER and an IPCE consistent with prior reports, we move on to the kinetics of photocurrent decay following illumination turn-off. In the capacitor ansatz described above the transient current after illumination turn-off is a consequence of recombination of holes stored in surface states under steady-state illumination. The two time scales apparent in the data and the H_2_O_2_ experiment suggest that the flow of electrons to the interface can be described as the charging of two, distinct, capacitors. Rewriting eqn (2) allows us to quantitatively describe such a situation,4
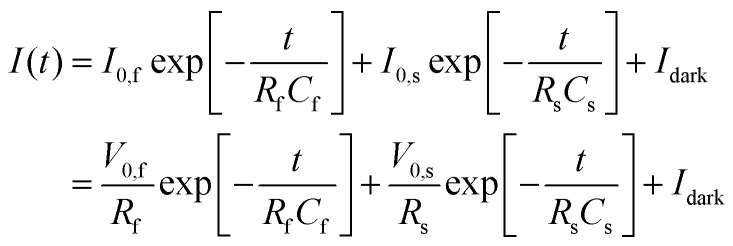
in which *I*_0,f_ and *I*_0,s_ are the currents flowing to the fast and slow relaxing surface states at time zero (*i.e.* under steady-state illumination conditions); *V*_0,f_ and *V*_0,s_ are the biases across the two capacitors at time zero; *R*_*i*_ and *C*_*i*_ are the resistances and capacitances associated with the *i*^th^ capacitor (either that associated with the fast or slow process) and *I*_dark_ is the dark current. Comparison of eqn (2) and (4) makes clear that *τ*_fast_ = *R*_f_*C*_f_ and *τ*_slow_ = *R*_s_*C*_s_. Because our two interfacial capacitors are in parallel, the bias across each fully charged is the externally applied bias minus the flat band potential (*V*_apl_ − *V*_fb_). The total charge stored in the *i*^th^ capacitor, *i.e. Q*_*i*_, is thus the integrated charge transferred to the capacitor associated with either the fast(f) or slow(s) relaxing process):5
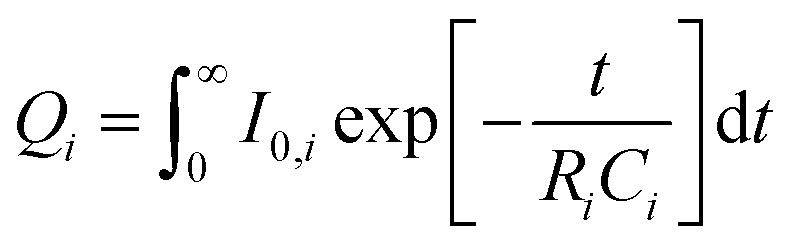
which we extract from numerically integrating the fit to the current transients. From the stored charge we compute the capacitances for the fast and slow processes: 
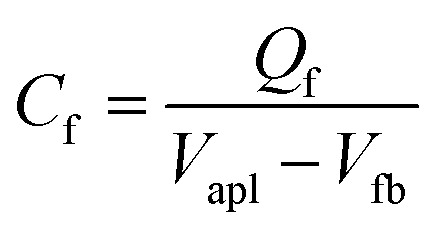
 and 
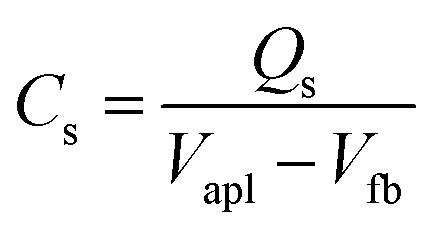
.

**Fig. 4:**
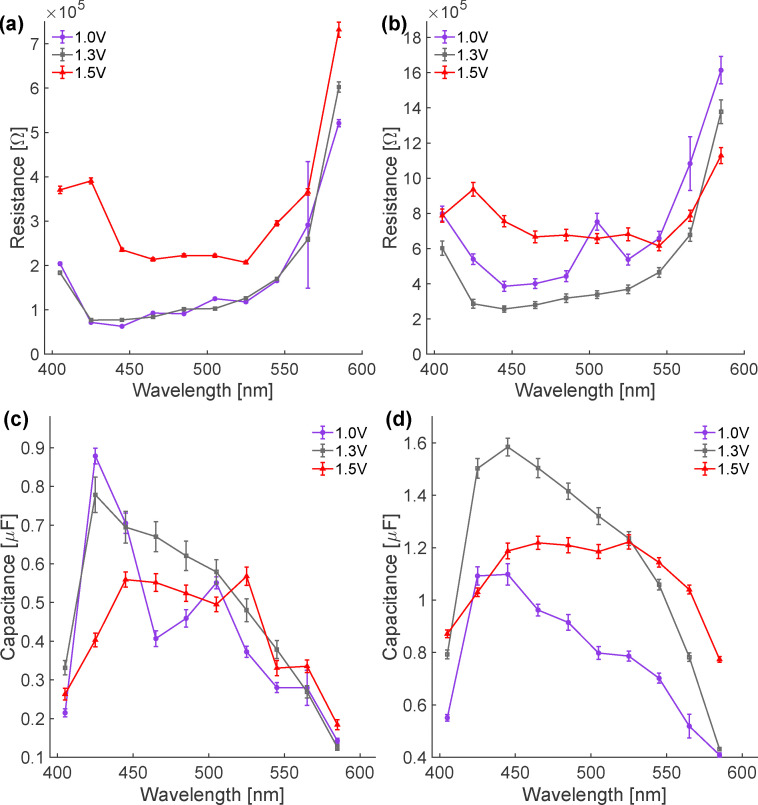
Extracted resistances and capacitances of the two surface states apparent in our chopped photocurrent data shown as a function of wavelength at biases below, near and above the oxygen evolution reaction onset. (a) Resistance associated with charging of monodentate coordinated surface O (*i.e.* the fast relaxing surface state); (b) resistance associated with the charging of bidentate coordinated surface O (*i.e.* the *slow* relaxing surface state); (c) capacitance of the monodentate coordinated surface. Data near 500 nm have larger uncertainty because the absolute transient amplitudes under pre-OER bias are small in this spectral region; (d) capacitance of the bidentate coordinated surface O. Error bars denote the standard deviations obtained from Monte-Carlo analysis of the transient fits.

Inspection of Fig. 4a and b shows that *R*_f_ and *R*_s_ below the OER onset (at 1.0 V *vs.* RHE) depend similarly on wavelength – somewhat higher at 405 nm, relatively low and constant between 445 and 525 nm and increasing at longer wavelengths. In principle the resistances we extract from fitting our data using eqn (4) may be a result of both transport through bulk and charge transfer at the interface. While the H_2_O_2_ data (see Fig. 2(c)) clearly show both the slow and fast processes are surface-mediated, the similarity in the wavelength dependence of *R*_f_ and *R*_s_ at potentials cathodic of OER onset, suggests the wavelength dependence of the resistances is the result of bulk hematite photophysics. Prior work has established that significant populations of excited electrons relax on < 100 fs timescales to form electron polarons in hematite.^18,39,40^ Leone and coworkers have shown that the character of the polaron changes depending on photon energy: at photon energies near the band gap the polaron is relatively localized and transport inefficient, near the absorption peak at ≈ 440 nm polarons are relatively delocalized and their mean free path longer.^39^ Our results appear to offer a similar perspective 
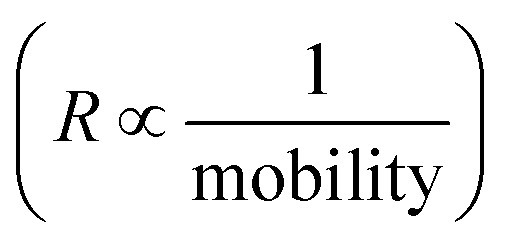
. Stated in the language of eqn (1)*η*_cs_ is a function of the physics of polarons in hematite. While the wavelength-dependence of *R*_f_ and *R*_s_ is similar, at all wavelengths *R*_s_ ≈ 3 × *R*_f_. This trend can be rationalized by accounting for the chemical nature of each surface state and the charge transfer required as it relaxes.

As noted above, imaging of our photoanode suggests it largely exposes the basal plane of hematite. Much prior work, both experimental and theoretical, on the hematite aqueous solution interface suggests that for the hematite basal plane the so-called 1-Fe and Oxygen terminated surfaces (there are three possible terminations for the α-Fe_2_O_3_(0001) surface^41^) are equally stable.^42–44^ As a consequence mixed surface terminations are often observed. For the oxygen terminated surface water adsorption results in bidentate coordinated surface oxygens, for the 1-Fe terminated, monodentate.^45^ Under practically relevant conditions the mono and bidentate oxygens are Lewis acids: the monodentate with a p*K*_a_ between 7–9, the bidentate > 12.^45,46^ Computational studies have clarified that illumination at potentials anodic of open circuit conditions results in the increased adsorption of OH^−^ on surface Fe atoms, *i.e.* for the 1-Fe terminated surface the number of monovalent coordinated OH increases, and the deprotonation of bivalent coordinated surface hydroxyl groups found on the oxygen terminated.^47^ Monodentate surface OH is expected to relax, on turning off illumination, by the desorption of OH^−^, bidentate O by the adsorption of H^+^.^47^ Because proton adsorption (from alkaline solution) is expected to be slow relative to OH^−^ desorption, a larger charge transfer resistance should be associated with the bidentate surface OH. We therefore conclude that our data are most simply understood if the fast relaxation process is assigned to charging of surface monodentate oxygens and the slow to charging of surface bidentate oxygens: *R*_f_ = *R*_mono_ and *R*_s_ = *R*_bi_. In the language of eqn (1) the difference in size of *R*_mono_ and *R*_bi_ suggests that *η*_ct_ contributes to both through differences in surface chemical speciation but that this contribution depends only weakly on the photon energy used to excite charge carriers between 450 and 550 nm (data at longer wavelength excitation are discussed further below).

In this scenario the charge relaxation processes we observe are present in the defect-free (0001) surface of α-Fe_2_O_3_. While we did not intentionally dope our hematite photoanode, much prior work suggests it should be n-type due to oxygen vacancies.^48^ Bulk oxygen vacancies increase electron concentration, modify the space-charge region and band bending, and thereby influence *J*_abs_, *ξ*_pg_, *η*_cs_ and the overall magnitude of the photocurrent, but they do not control the time constants extracted from eqn (4) (recall that H_2_O_2_ experiments suggest these processes are surface-mediated).^48^ In contrast, oxygen vacancies near, but not at, the surface directly perturb the first few Fe–O layers and leave under-coordinated Fe sites at the interface.^49^ Prior theoretical studies offer a mixed view as to whether such structures lead to enhanced or decreased OER activity (based in part on model chemistry employed or OER mechanism assumed).^50,51^ Our measured photocurrent kinetics do not allow us to separate any possible effect of near surface oxygen vacancies on our observables. However, prior work strongly suggests that two different types of surface OH, *i.e.* two different types of surface electronic states, are likely present on the pristine hematite/aqueous solution interface,^47^ and that we therefore need not invoke defects to rationalize our observations.

Given this assignment, we return to the data plotted in Fig. 4 and note that the *R*_mono_ does not vary as the bias is increased from potentials cathodic to those at the OER onset, *i.e.* at 1.0 and 1.3 V *vs.* RHE. In contrast, while the wavelength-dependence is similar, *R*_bi_ increases by ≈20% going from 1.0 to 1.3 V *vs.* RHE. This increase suggests an additional charge transfer resistance has been added to bidentate surface oxygens near the OER onset that does not depend on photon energy. Prior workers have argued from computational results that bidentate surface oxygens are likely the most active site for the light induced OER.^8,52^ These results are consistent with our observations if, as assigned above, the slow process is a consequence of (dis)charging of bidentate surface oxygen sites. At 1.5 V both *R*_mono_ and *R*_bi_ increase further, albeit with decrease in *R*_bi_ at long wavelength. As reference to Fig. 1 makes clear, at 1.5 V *vs.* RHE the OER has begun in the absence of illumination. Our measured *R*_mono_ and *R*_bi_ suggest that at these elevated potentials the OER mechanism is potential-dependent: monodentate coordinated surface sites are activated and/or a multihole OER mechanism on hematite may be possible.^8,12,25,53^

In the surface capacitor ansatz *C*_mono_ and *C*_bi_ are proportional to charge storage capacity. The wavelength-dependent *C*_mono_ and *C*_bi_ are shown at 1.0, 1.3 and 1.5 V *vs.* RHE in Fig. 4(c) and (d). At 1 V *vs.* RHE clearly both sites show a peak in capacitance at 420–450 nm that decreases towards 580 nm with *C*_bi_ showing a shoulder at ≈500 nm. At 1.3 V *vs.* RHE *C*_mono_ is similar to its value at 1.0 V *vs.* RHE with a possible decrease in capacitance between 450 and 550 nm. Over the same change in bias *C*_bi_ increases by ≈50% at all photon energies. These observations are consistent with a scenario in which, at 1.3 V *vs.* RHE, the OER begins on bidentate coordinated surface oxygens and steady-state populations of more oxidized oxygen species are present at the interface. At 1.5 V *vs.* RHE the wavelength-dependent response of both *C*_mono_ and *C*_bi_ are similar with the shoulder centered at 500 nm continuing to grow in for both species. This correlated change in capacitance is consistent with a scenario in which OER mechanism is potential-dependent and at these anodic biases involves both mono and bidentate coordinated surface OH.

**Fig. 5:**
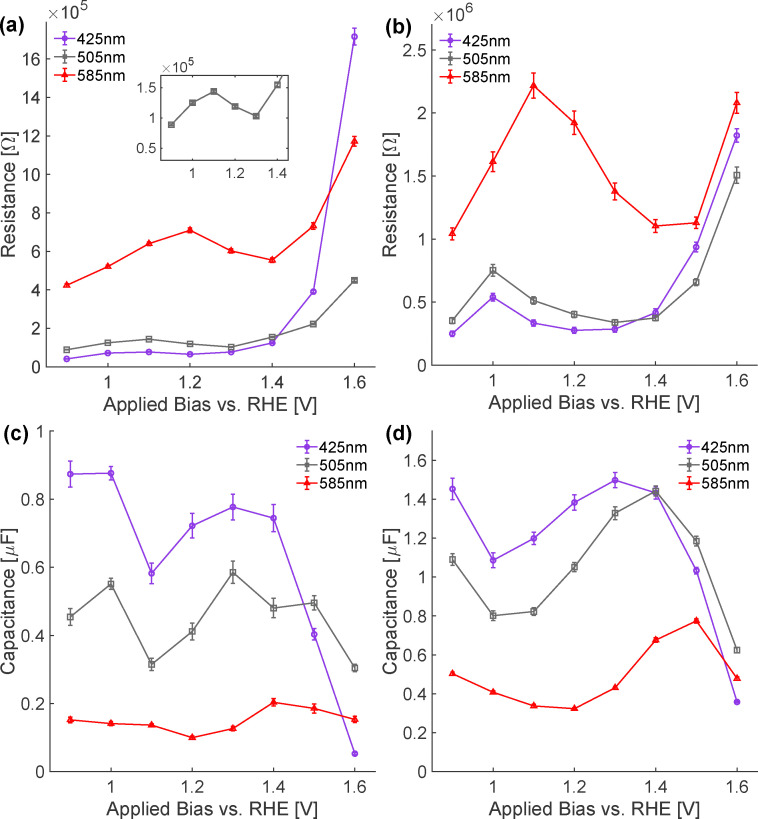
Bias dependence of the surface-state resistances and capacitances at three excitation wavelengths. (a) *R*_mono_ and (b) *R*_bi_ at 425, 505 and 585 nm as a function of applied potential *vs.* RHE. (c) *C*_mono_ and (d) *C*_bi_ at the same wavelengths and potentials. For 425 and 505 nm, *R*_bi_ exhibits a maximum at more cathodic bias than *R*_mono_, while the minima in *C*_bi_ are shifted relative to those in *C*_mono_ similarly. Both observations are consistent with a scenario in which bidentate surface OH groups are catalytically active for the OER while monodentate OH respond to bias to regulate changing surface charge. Excitation at 585 nm leads to substantially larger resistances and smaller capacitances for both manifolds, consistent with the low IPCE and the limited population of surface states when holes are generated predominantly *via* d–d transitions. Error bars denote the standard deviations obtained from Monte-Carlo analysis of the transient fits.

Because we expect to see a maximum in *R*, *i.e.* a maximum in charge transfer resistance, just before current starts to flow, we next examine the bias dependence of *R*_mono_ and *R*_bi_ with higher bias resolution (see Fig. 5). Employing excitations of 425 and 505 nm, *R*_mono_ has a small maximum at 1.1 V and at potentials greater than 1.4 V *vs.* RHE increases dramatically. In contrast *R*_bi_ has a somewhat larger maximum at 1.0 V *vs.* RHE and increases significantly above 1.4 V. The maximum in *R*_bi_ cathodic of and larger than that in *R*_mono_ suggests that bidentate surface coordinated OH are the active site for photoinduced OER. Given the band edge pinning suggested by the impedance measurements discussed above, the increase in *R*_mono_ following the onset of the OER is most straightforwardly understood as either surface protonation or OH^−^ adsorption required to ensure bias-independent surface charge density. Large increases in *R*_mono_ and *R*_bi_ at higher biases suggest as OER in the dark becomes possible monodentate OH may participate in the OER and/or additional OER mechanisms become possible.

The corresponding capacitances provide complementary information on how the interfacial hole population is distributed between the two surface site types (Fig. 5(c) and (d)). For 425 and 505 nm excitation, *C*_mono_ and *C*_bi_ both show a non-monotonic bias dependence: starting from low bias they first decrease, then increase, and finally drop again at the highest potentials. Importantly, however, the positions of the extrema differ for the two surface state populations. In particular, the minimum in *C*_bi_ occurs at a slightly more cathodic bias than the minimum in *C*_mono_, and *C*_bi_ starts to rise while *C*_mono_ is still decreasing. This behavior mirrors the trend observed in the resistances, where the maximum in *R*_bi_ appears cathodic of that in *R*_mono_.

On the atomic level the initial decrease of *C*_mono_ and *C*_bi_ can be understood as the protonation of existing surface oxygens as the space-charge region develops. The subsequent increase in capacitance around the minima reflects the build-up of hole charge in the respective surface site manifolds: for *C*_mono_ this corresponds to a growing reservoir of monodentate Fe–OH groups that buffers the interfacial charge, whereas the rise in *C*_bi_ marks the accumulation of OER intermediates on bidentate Fe–O(H) species. The fact that *C*_bi_ begins to grow at more cathodic potentials, in parallel with the cathodic maximum in *R*_bi_, is most simply explained if bidentate surface oxygens accumulate catalytically relevant holes, and experience a reduction in charge-transfer resistance cathodically of the potential-dependent monodentate oxygen response. The final decrease of both capacitances at the highest biases, together with the large values of *R*_mono_ and *R*_bi_ in this regime, suggests that monodentate OH may participate in the OER and/or additional OER mechanisms become possible.

Rationalizing both the bias-dependent *R*_mono_ and *R*_bi_ following 585 nm excitation is more challenging. Much prior work has noted that the absorption spectrum of hematite is the result of two ligand to metal charge transfer (LMCT) transitions (a stronger O 2p → e_g_ in-plane transition centered at ≈250 nm and a weaker O 2p → t_2g_ transition at ≈400 nm) and a series of d–d transitions that dominate the absorption between the LMCT transitions, at ≈350 nm, and at long wavelengths (where the LMCT excitation results in an O centered hole and the d–d an Fe).^16,17,54,55^ Quantitative modeling of the IPCE suggests that excitations of d–d transitions are substantially less catalytically active than those of LMCT.^17^*R*_mono_ and *R*_bi_ resulting from 585 nm excitation have a qualitatively similar bias dependence that is shifted anodically and much larger, <1.5 V *vs.* RHE, than with excitation at smaller wavelengths. Similarly *C*_mono_ and *C*_bi_ are substantially smaller than at 425 and 505 nm over the entire bias range, indicating that when excitation proceeds predominantly *via* d–d transitions far fewer holes reach and are stored in either surface-state manifold.

The increase in both *R*_mono_ and *R*_bi_ employing 585 nm excitation relative to lower wavelengths suggests that, in addition to the trend in electron polaron mobility observed by Leone and coworkers discussed above,^39^ the different hole types likely have different mobilities in bulk hematite and that, as observed by Braun and coworkers^54^ in *operando* x-ray absorption measurements, both populations are affected by OER onset. As discussed above many structural studies have clarified that mono- and bidentate surface hydroxyls may be present on the α-Fe_2_O_3_(0001) surface in contact with liquid H_2_O in the absence of illumination or applied bias. As shown in Fig. 5, 585 nm illumination results in an added series resistance to both *R*_mono_ and *R*_bi_. The fact that, when pumping with 585 nm, a maximum in *R*_bi_ appears cathodic of a maximum in *R*_mono_ but that both are shifted with respect to *R*_bi_ and *R*_mono_ at shorter wavelengths, suggests, consistent with the argument of Braun *et al.*, that d–d type hole populations are bias-dependent: they appear to induce deprotonation of bidentate OH and adsorption of monodentate OH similarly to LMCT excitations. The anodic shift in bias, however, suggests that an additional elementary process, such as a conversion of hole type, follows illumination at these wavelengths. *Operando* X-Ray absorption measurements conducted as a function of bias and wavelength (using monochromatic illumination) would be extremely useful in understanding this point.

The OER mechanism on hematite has been studied by multiple groups both experimentally and theoretically. At the illumination powers employed in this experiment‡‡The incident photon flux density is ≈9.4 × 10^16^ cm^−2^ s^−1^, while for the AM 1.5G spectrum integrated over 405–645 nm it is 9.3 × 10^16^ cm^−2^ s^−1^. Note, though, that the AM 1.5G spectrum spans a much broader wavelength range, extending from roughly 300 nm to the mid-infrared region.^56^ a four step proton coupled electron transfer (PCET) mechanism is generally thought to occur in alkaline solution (generally stated without regard for the coordination of the surface O atom)^29,57^

6 Fe* + OH^−^ + h^+^ ↔ Fe–OH

7 Fe–OH + OH^−^ + h^+^ ↔ Fe

<svg xmlns="http://www.w3.org/2000/svg" version="1.0" width="13.200000pt" height="16.000000pt" viewBox="0 0 13.200000 16.000000" preserveAspectRatio="xMidYMid meet"><metadata>
Created by potrace 1.16, written by Peter Selinger 2001-2019
</metadata><g transform="translate(1.000000,15.000000) scale(0.017500,-0.017500)" fill="currentColor" stroke="none"><path d="M0 440 l0 -40 320 0 320 0 0 40 0 40 -320 0 -320 0 0 -40z M0 280 l0 -40 320 0 320 0 0 40 0 40 -320 0 -320 0 0 -40z"/></g></svg>


O + H_2_O

8 FeO + OH^−^ + h^+^ ↔ Fe–OOH

9 Fe–OOH + OH^−^ + h^+^ ↔ Fe⋯O_2_(ad) + H_2_O

10 Fe–O_2_(ad) → O_2_ + Fe*

Whether a surface O vacancy exists on α-Fe_2_O_3_ under operando conditions is unclear: in general most results suggest that adsorption of H_2_O or OH^−^ is thermodynamically favored and rapid at bidentate sites but not monodentate. Regardless of this point, however, most computational studies suggest that either reaction (7)^12,52^ or reaction (8)^58,59^ are rate limiting while experiment suggests reaction (8).^60,61^ Our observations here are consistent with a scenario in which photoinduced OER proceeds through bidentate surface OH but suggest that, as the faradaic current associated with the OER begins, additional holes are stored at the interface through adsorption of OH^−^ and formation of monodentate coordinated surface hydroxyls (*i.e.*reaction (6)).

Noh *et al.* recently found in a computational study that thermodynamically plausible OER mechanisms exist on both the 1-Fe and O terminated α-Fe_2_O_3_(0001) surfaces.^47^ In the case of the monodentate coordinated oxygen the initial step in OER is adsorption of an OH^−^, in the case of the bidentate the desorption of a surface proton. While important this study does not consider the surface protonation state (monodentate surface OH are expected to be significantly deprotonated under alkaline open-circuit conditions, bidentate not), its bias-dependent change, and the manner in which surface protonation might be expected to influence OER kinetics. The additional protonation step required for this mechanism to apply for monodentate coordinated surface oxygens rationalizes our suggestion that under *operando* conditions OER proceeds through bidentate coordinated surface hydroxyls on the hematite basal plane.

It is worth emphasizing that even if the OER is dominated by bidentate surface hydroxyls, this does not imply that the monodentate surface oxygen protonation state or population are independent of applied cathodic bias or illumination. Textbook treatments suggest that at potentials cathodic of OER onset, in the absence of Fermi level pinning, increasing bias would lead to surface protonation (as a means of charge compensation) while under illumination one might expect additional adsorption of OH^−^ (as a means of hole storage).^47^ Unravelling the interaction between surface termination, surface protonation state and OER activity requires interface-specific, *operando*, long-wavelength vibrational spectroscopy of the α-Fe_2_O_3_/H_2_O interface.^62,63^ Such studies are underway in our group.

**Scheme 1:**
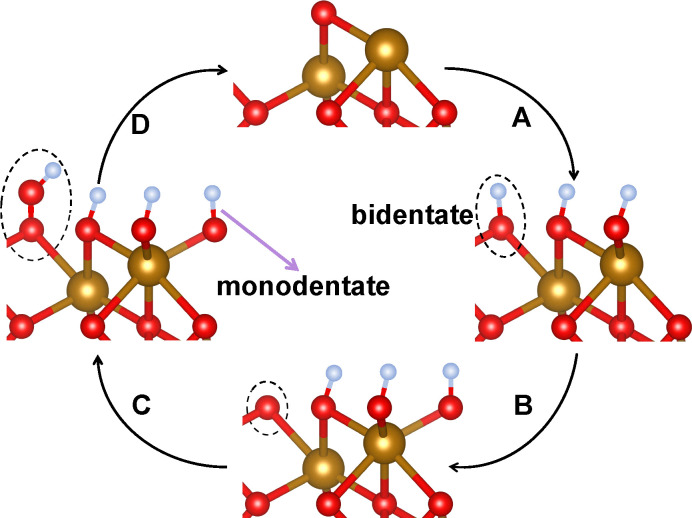
Illustration of OER mechanism on hematite. Gold spheres denote Fe, red spheres O, and blue spheres H. The pathway A → B → C → D corresponds to bidentate coordinated Fe–O(H) species (marked by dashed ellipses) that host the catalytic OER intermediates: A: formation of a hole-bearing Fe–OH unit (reaction (6)); B: further oxidation of bidentate OH to Fe=O (reaction (7)); C: nucleophilic attack of OH^−^ to form Fe–OOH (reaction (8)); D: O_2_ release and regeneration of Fe* (reaction (9), followed by step (10), not explicitly shown). The bias-dependence of resistances and capacitance is consistent with an (0001) surface characterized by both monodentate and bidentate OH. Bias-dependent measurements of current transients suggest, consistent with theoretical prediction, that the bidentate OH is catalytically active and that the monodentate population adjusts to compensate for surface hole population as the catalytic cycle begins.

Stated in the language of eqn (1) these measurements allow us to separate the surface and bulk controls on the wavelength-dependent *J*_photo_. The similar, and relatively bias-independent, wavelength-dependence of both *R*_mono_ and *R*_bi_ suggest that these parameters are largely controlled by the bulk transport properties of electrons and holes as well as the hematite optical transition excited: they directly characterize *η*_cs_. As described above, *η*_ct_ is expected to be dominated by surface chemical transformations. The bias dependence of *R*_mono_ and *R*_bi_ suggest that the OER proceeds through bidentate coordinated surface oxygen but that monodentate coordinated surface sites (re)adjust to compensate surface polarization. Surprisingly, even above OER onset, the wavelength dependence of *R*_mono_ and *R*_bi_ is similar. We take this observation to suggest that, contrary to expectation, the OER is relatively insensitive to the nature of the hole that reaches the surface: charge transfer resistance, which accumulates as increasing numbers of holes are stored on the surface and the OER begins, is independent of photon energy below ≈570 nm. At wavelengths above ≈570 nm, where d–d excitations dominate over LMCT, an additional resistance appears for both *R*_mono_ and *R*_bi_: Fe centered holes can be stored in both types of surface hydroxyls at additional energetic cost. The scenario is sketched in Scheme 1. Taken as a whole, these conclusions highlight the necessity in understanding surface protonation state and OH population as a function of bias and in the presence of illumination in understanding the OER mechanism on hematite and other metal oxides.

## 4. Conclusions

Understanding the elementary processes controlling surface hole accumulation and loss is important if we are ever to build the best possible semiconducting photoanodes in general and α-Fe_2_O_3_ photoanodes in particular. Steady-state photocurrent measurements, so-called IPCE spectra, convolute the relevant elementary processes making it challenging to understand, in the absence of additional information, whether loss of absorbed photons results from recombination in bulk or surface-mediated processes. Bias-dependent photocurrent kinetics measurements on millisecond to second timescales in principle offer a means of separating these effects. Here we show, by collecting current-off kinetics as a function of applied bias and wavelength of illumination, that our α-Fe_2_O_3_(0001) surface has both monodentate and bidentate coordinated surface hydroxyls where OER proceeds through the latter but the former responds to regulate surface charge. Holes produced in bulk α-Fe_2_O_3_ populate both types of surface sites similarly. This observation extends even to relatively unreactive long wavelength d–d type excitations. Here, however, an additional resistance suggests hole conversion may be required in order to induce OER activity.

This relatively simple approach appears to offer access to the elementary processes underlying observations of strong wavelength dependence in *J*_photo_. Comparing similar measurements α-Fe_2_O_3_ doped or with co-deposited catalysts would offer clear insights into the mechanism by which hematite electrode modifications affect OER activity and selectivity.

## Author contributions

YY: conceptualization, data curation, formal analysis, investigation, methodology, software, visualization. FZ: investigation, formal analysis, software, visualization. SS and HW: resources, investigation. GB and SS: investigation. YT: conceptualization, formal analysis, methodology, supervision. RKC: conceptualization, funding acquisition, methodology, project administration, supervision. YY and RKC: writing – original draft. All authors: writing – review and editing.

## Conflicts of interest

There are no conflicts to declare.

## Supplementary Material

CP-028-D5CP04300J-s001

## Data Availability

Supplementary information: Raman and XRD spectra, SEM image, the electrochemical cell schematic, EIS data with fitted capacitances, photocurrent characteristics under CA as a function of illumination power, bias-dependent resistances and capacitances measured at different wavelengths in the dark, the data-processing workflow with flow chart and Monte Carlo uncertainty analysis, Mott–Schottky analysis with space-charge layer thickness estimation, and TMM-based absorptance calculations. See DOI: https://doi.org/10.1039/d5cp04300j. All data that appears in this paper and the supplementary information are available in the RESOLVdata repository; DOI: 10.17877/RESOLV-2026-U3ON8W.

## References

[cit1] Hardee K. L., Bard A. J. (1976). J. Electrochem. Soc..

[cit2] Marusak L. A., Messier R., White W. B. (1980). J. Phys. Chem. Solids.

[cit3] Kennedy J. H., Frese K. W. (1978). J. Electrochem. Soc..

[cit4] Sivula K., Le Formal F., Grätzel M. (2011). ChemSusChem.

[cit5] Le Formal F., Pendlebury S. R., Cornuz M., Tilley S. D., Grätzel M., Durrant J. R. (2014). J. Am. Chem. Soc..

[cit6] Gärtner W. W. (1959). Phys. Rev..

[cit7] Tilley S. D., Cornuz M., Sivula K., Grätzel M. (2010). Angew. Chem., Int. Ed..

[cit8] Righi G., Plescher J., Schmidt F.-P., Campen R. K., Fabris S., Knop-Gericke A., Schlögl R., Jones T. E., Teschner D., Piccinin S. (2022). Nat. Catal..

[cit9] Kay A., Cesar I., Grätzel M. (2006). J. Am. Chem. Soc..

[cit10] Grave D. A., Dotan H., Levy Y., Piekner Y., Scherrer B., Malviya K. D., Rothschild A. (2016). J. Mater. Chem. A.

[cit11] Su J., Wang J., Liu C., Feng B., Chen Y., Guo L. (2016). RSC Adv..

[cit12] Liao P., Keith J. A., Carter E. A. (2012). J. Am. Chem. Soc..

[cit13] Kim J. Y., Magesh G., Youn D. H., Jang J.-W., Kubota J., Domen K., Lee J. S. (2013). Sci. Rep..

[cit14] Grave D. A., Ellis D. S., Piekner Y., Kölbach M., Dotan H., Kay A., Schnell P., Van De Krol R., Abdi F. F., Friedrich D., Rothschild A. (2021). Nat. Mater..

[cit15] Piekner Y., Ellis D. S., Grave D. A., Tsyganok A., Rothschild A. (2021). Energy Environ. Sci..

[cit16] Chernyshova I. V., Ponnurangam S., Somasundaran P. (2010). Phys. Chem. Chem. Phys..

[cit17] Hayes D., Hadt R. G., Emery J. D., Cordones A. A., Martinson A. B. F., Shelby M. L., Fransted K. A., Dahlberg P. D., Hong J., Zhang X., Kong Q., Schoenlein R. W., Chen L. X. (2016). Energy Environ. Sci..

[cit18] Pastor E., Park J.-S., Steier L., Kim S., Grätzel M., Durrant J. R., Walsh A., Bakulin A. A. (2019). Nat. Commun..

[cit19] Dotan H., Sivula K., Grätzel M., Rothschild A., Warren S. C. (2011). Energy Environ. Sci..

[cit20] Barroso M., Mesa C. A., Pendlebury S. R., Cowan A. J., Hisatomi T., Sivula K., Grätzel M., Klug D. R., Durrant J. R. (2012). Proc. Natl. Acad. Sci. U. S. A..

[cit21] Le Formal F., Sivula K., Grätzel M. (2012). J. Phys. Chem. C.

[cit22] Peter L. M., Wijayantha K. G. U., Tahir A. A. (2012). Faraday Discuss..

[cit23] Cummings C. Y., Marken F., Peter L. M., Tahir A. A., Wijayantha K. G. U. (2012). Chem. Commun..

[cit24] Klotz D., Ellis D. S., Dotan H., Rothschild A. (2016). Phys. Chem. Chem. Phys..

[cit25] Le Formal F., Pastor E., Tilley S. D., Mesa C. A., Pendlebury S. R., Grätzel M., Durrant J. R. (2015). J. Am. Chem. Soc..

[cit26] Cesar I., Sivula K., Kay A., Zboril R., Grätzel M. (2009). J. Phys. Chem. C.

[cit27] Peerakiatkhajohn P., Yun J.-H., Chen H., Lyu M., Butburee T., Wang L. (2016). Adv. Mater..

[cit28] Dotan H., Kfir O., Sharlin E., Blank O., Gross M., Dumchin I., Ankonina G., Rothschild A. (2013). Nat. Mater..

[cit29] George K., Khachatrjan T., Van Berkel M., Sinha V., Bieberle-Hütter A. (2020). ACS Catal..

[cit30] Klahr B., Gimenez S., Fabregat-Santiago F., Hamann T., Bisquert J. (2012). J. Am. Chem. Soc..

[cit31] Lohaus C., Klein A., Jaegermann W. (2018). Nat. Commun..

[cit32] Hankin A., Bedoya-Lora F. E., Alexander J. C., Regoutz A., Kelsall G. H. (2019). J. Mater. Chem. A.

[cit33] Iandolo B., Zhang H., Wickman B., Zoric I., Conibeer G., Hellman A. (2015). RSC Adv..

[cit34] Emin S., de Respinis M., Mavric T., Dam B., Valant M., Smith W. A. (2016). Appl. Catal., A.

[cit35] Grave D. A., Segev G. (2022). J. Phys. D: Appl. Phys..

[cit36] Glasscock J. A., Barnes P. R. F., Plumb I. C., Savvides N. (2007). J. Phys. Chem. C.

[cit37] Piccinin S. (2019). Phys. Chem. Chem. Phys..

[cit38] Chen C. T., Cahan B. D. (1981). J. Opt. Soc. Am..

[cit39] Carneiro L. M., Cushing S. K., Liu C., Su Y., Yang P., Alivisatos A. P., Leone S. R. (2017). Nat. Mater..

[cit40] Biswas S., Husek J., Londo S., Baker L. R. (2018). Nano Lett..

[cit41] Kurita T., Uchida K., Oshiyama A. (2010). Phys. Rev. B:Condens. Matter Mater. Phys..

[cit42] Wang X. G., Weiss W., Shaikhutdinov S. K., Ritter M., Petersen M., Wagner F., Schlögl R., Scheffler M. (1998). Phys. Rev. Lett..

[cit43] Trainor T. P., Chaka A. M., Eng P. J., Newville M., Waychunas G. A., Catalano J. G., Brown Jr. G. E. (2004). Surf. Sci..

[cit44] Kiejna A., Pabisiak T. (2013). J. Phys. Chem. C.

[cit45] Gittus O. R., von Rudorff G. F., Rosso K. M., Blumberger J. (2018). J. Phys. Chem. Lett..

[cit46] Lützenkirchen J., Preocanin T., Stipic F., Heberling F., Rosenqvist J., Kallay N. (2013). Geochim. Cosmochim. Acta.

[cit47] Noh J., Li H., Osman O. I., Aziz S. G., Winget P., Brédas J.-L. (2018). Adv. Energy Mater..

[cit48] Fan Z., Wen X., Yang S., Lu J. G. (2005). Appl. Phys. Lett..

[cit49] Boily J.-F., Chatman S., Rosso K. M. (2011). Geochim. Cosmochim. Acta.

[cit50] Nguyen M.-T., Piccinin S., Seriani N., Gebauer R. (2015). ACS Catal..

[cit51] Wang R. B., Hellman A. (2019). J. Phys. Chem. C.

[cit52] Nguyen M.-T., Seriani N., Piccinin S., Gebauer R. (2014). J. Chem. Phys..

[cit53] Mesa C. A., Francàs L., Yang K. R., Garrido-Barros P., Pastor E., Ma Y., Kafizas A., Rosser T. E., Mayer M. T., Reisner E., Grätzel M., Batista V. S., Durrant J. R. (2020). Nat. Chem..

[cit54] Braun A., Sivula K., Bora D. K., Zhu J., Zhang L., Grätzel M., Guo J., Constable E. C. (2012). J. Phys. Chem. C.

[cit55] Liao P., Carter E. A. (2011). J. Phys. Chem. C.

[cit56] Gueymard C. A., Myers D., Emery K. (2002). Sol. Energy.

[cit57] Hellman A., Pala R. G. S. (2011). J. Phys. Chem. C.

[cit58] Yatom N., Neufeld O., Caspary Toroker M. (2015). J. Phys. Chem. C.

[cit59] Yatom N., Elbaz Y., Navon S., Toroker M. C. (2017). Phys. Chem. Chem. Phys..

[cit60] Zandi O., Hamann T. W. (2016). Nat. Chem..

[cit61] Li J., Chen H., Liu S., Wang Y., Wan W., Zhao Y., Triana C. A., Xu Z., Patzke G. R. (2025). J. Am. Chem. Soc..

[cit62] Tong Y., Wirth J., Kirsch H., Wolf M., Saalfrank P., Campen R. K. (2015). J. Chem. Phys..

[cit63] Yue Y., Melani G., Kirsch H., Paarmann A., Saalfrank P., Campen R. K., Tong Y. (2022). J. Phys. Chem. C.

