# Supplementary material for: Characterizing two surface states and their role in the photoinduced oxygen evolution reaction on hematite via photocurrent kinetics
Source: Phys Chem Chem Phys. 2026 Mar 12;28(15):9315–25. doi: 10.1039/d5cp04300j (PMC13020682; doi:10.1039/d5cp04300j)
Supplement: CP-028-D5CP04300J-s001 [file CP-028-D5CP04300J-s001.pdf]

Supplementary Information (SI) for Physical Chemistry Chemical Physics.
This journal is © the Owner Societies 2026

Supplementary Information: Characterizing two surface states and their role in the photoinduced oxygen evolution reaction on hematite via photocurrent kinetics

Yuke Yang[1], Felix Zerres[1], Soma Salamon[1,2], Georg Bendt[3], Stephan Schulz[3], Heiko Wende[1,2], Yujin Tong[1], and R. Kramer Campen[1,*]

[1]Faculty of Physics, University of Duisburg-Essen, Lotharstr. 1, 47057 Duisburg, Germany
[2]Faculty of Chemistry, University of Duisburg-Essen, Universitätsstr. 7, 45141 Essen, Germany
[3]Center for Nanointegration Duisburg-Essen (CENIDE), University of Duisburg-Essen, Lotharstr. 1, 47057 Duisburg, Germany
[*]e-mail: richard.campen@uni-due.de

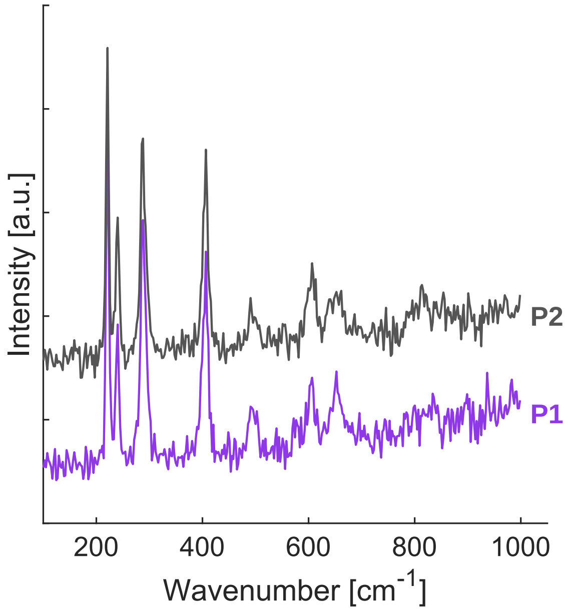

The spectra therefore confirm the phase purity of the hematite film without detectable impurities of other iron oxide phases.

Figure S1: Raman spectra of post-annealing hematite sample. Spectra were acquired using a WITec alpha300 RA confocal Raman spectrometer with a 532.328 nm green laser excitation source, a 600 grooves/mm diffraction grating and a laser power of 2 mW. P1 and P2 are different detection positions. As reported in prior studies, hematite (α-Fe_2O_3) exhibits two A_{1g} (225 and 498 cm^{-1}) and five E_g (247, 293, 299, 412, and 613 cm^{-1}) Raman-active modes, while γ-Fe_2O_3 and Fe_3O_4 lack these features.[1] A band near 650 cm^{-1}, frequently observed for hematite photoanodes, has been attributed to either minor iron-oxide impurities or defect-related scattering (e.g., dopants/structural disorder).[2] Here, no additional peaks attributable to γ-Fe_2O_3 or Fe_3O_4 are detected across the measured range; thus, the $\sim$650 cm^{-1} band is assigned to defect-activated scattering within hematite, where lattice disorder (such as oxygen vacancies and local strain) relaxes Raman selection rules and enhances otherwise weak phonon contributions.

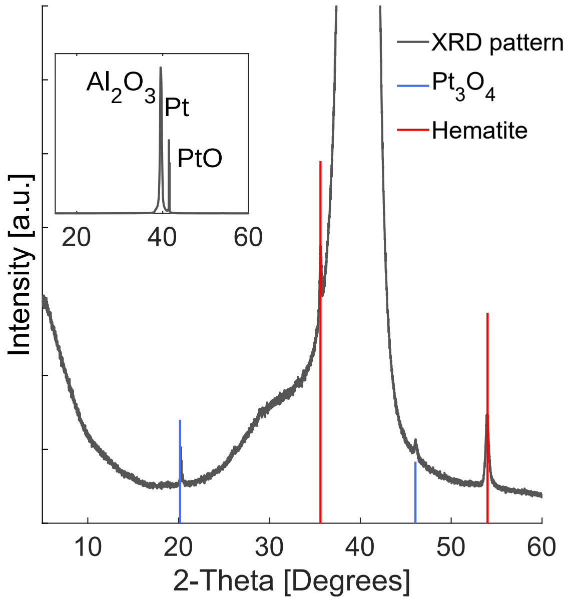

Figure S2: XRD pattern of the annealed hematite anode. It shows a magnified view of the measured XRD pattern (inset), highlighting the reflections. Data were collected on a Bruker D8 diffractometer with Cu Kα radiation (λ = 1.5406 Å) over $2\theta = 5 - 60°$ with a step size of $0.01°$. The black trace is the measured pattern; red sticks mark the reference peaks of hematite (PDF $\#72 - 0469$), and blue stick the reference peak of Pt_3O_4 (PDF $\#74 - 1870$). Reference peaks of the substrates are indexed to Al_2O_3 (PDF $\#10 - 0425$), Pt (PDF $\#04 - 0802$) and PtO (PDF $\#42 - 0866$), respectively. No reflections attributable to other iron-oxide phases are observed when compared with their PDF reference patterns, indicating high phase purity of hematite.

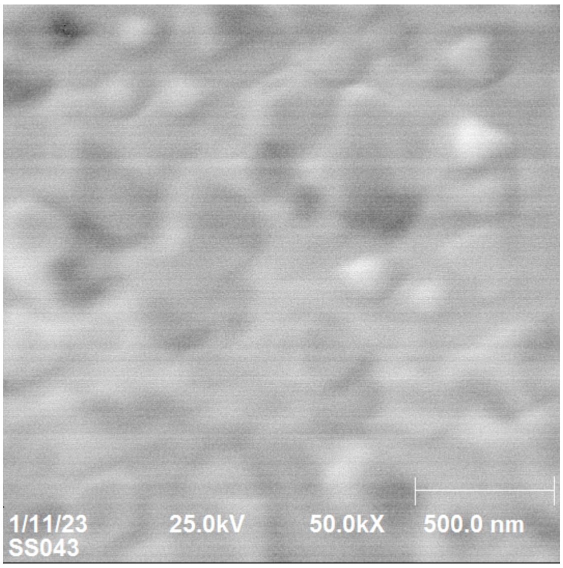

Figure S3: SEM image of hematite sample post-annealing collected using a PHI 710 NanoProbe instrument (Ulvac-Phi) operated at 25 kV with a sub-6 nm electron beam diameter. The sample exhibits a well-crystallized morphology, with grains predominantly exposing the (0001) facet. For hematite, as for all corundum-type minerals, the triangles are (0001) terraces viewed normal to the c-axis[3;4].

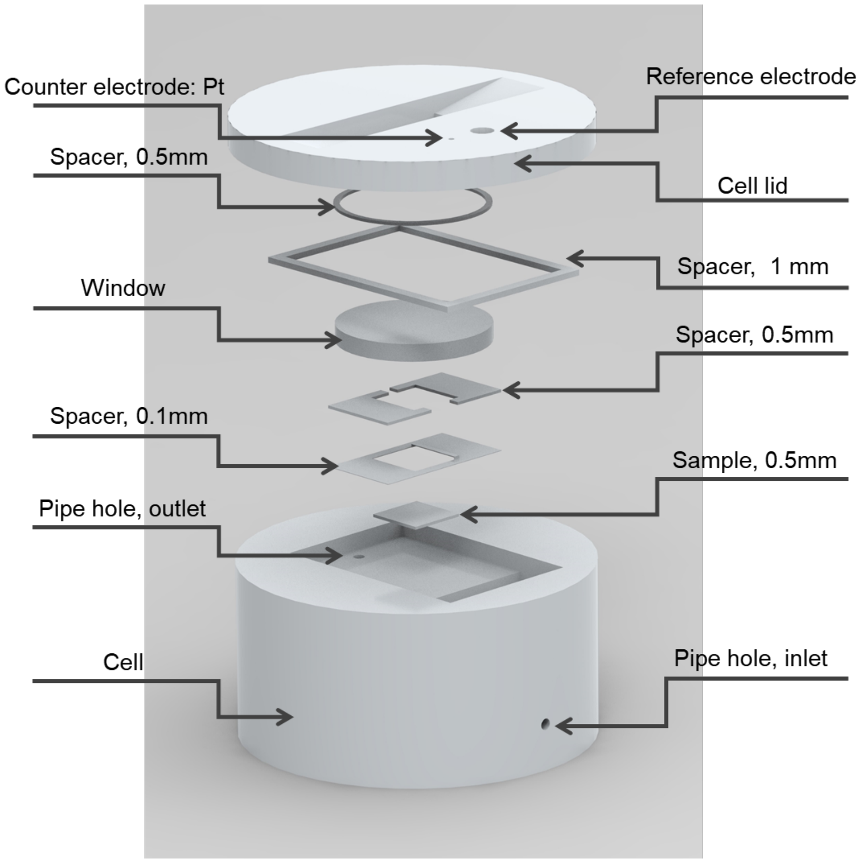

Figure S4: Schematic of the home-made Teflon electrochemical cell. The annotated values indicate the thicknesses of the sample and each spacer layer. The cell is equipped with PTFE spacers, a CaF_2 optical window, a Pt counter electrode and an Ag/AgCl reference electrode. Considering the cumulative thickness of all spacers, as well as the copper tape for electrical contact and Teflon tape used to seal the sample edges, the resulting electrolyte layer is $\approx 200\mu$m thick.

Figure S5 summarizes the data-processing workflow used to obtain the quantities shown in Figures 3–5 in the manuscript. For each potential and wavelength, a linear baseline was first determined by fitting the dark current in an 8 s window before the first light pulse and an 8 s window after the last pulse. This baseline was subtracted from the raw current trace, and the three consecutive pulses in the baseline-corrected data were then averaged to yield a single representative transient. The resulting photocurrent $I(t)$ in the light-on and light-off segment was then fitted to a bi-exponential + offset,

$$I(t) = A\exp\left(-\frac{t}{\tau_{\text{fast}}}\right) + B\exp\left(-\frac{t}{\tau_{\text{slow}}}\right) + I_{\text{ss}} \tag{1}$$

using the `nlinfit` nonlinear least-squares algorithm (MATLAB R2023b). Here A and B are the amplitudes of the fast and slow decays, τ_{fast} and τ_{slow} are the corresponding time constants, and I_{ss} is the steady-state photocurrent under illumination or dark current for the light-off transient. The steady-state values $I_{\text{ss}}(\lambda)$ under illumination were also used to compute the wavelength-dependent IPCE,

$$\text{IPCE}(\lambda) = \frac{I_{\text{ss}}(\lambda)}{e\,N_{\text{photon}}(\lambda)}, \tag{2}$$

where $N_{\text{photon}}(\lambda)$ is the calibrated incident photon flux at the sample.

From the fitted amplitudes and time constants, the charges associated with the fast and slow surface-state populations were obtained by numerical integration of the exponential terms over the experimentally relevant time window $t = 0 - 4$ s, using the sampling interval $\Delta t = 0.02$ s:

$$Q_{\text{fast}} = \sum_{t=0}^{4\text{ s}} A\exp\left(-\frac{t}{\tau_{\text{fast}}}\right)\Delta t, \qquad Q_{\text{slow}} = \sum_{t=0}^{4\text{ s}} B\exp\left(-\frac{t}{\tau_{\text{slow}}}\right)\Delta t. \tag{3}$$

The corresponding capacitances were defined as

$$C_{\text{fast}} = \frac{Q_{\text{fast}}}{V_{\text{appl}} - V_{\text{fb}}}, \qquad C_{\text{slow}} = \frac{Q_{\text{slow}}}{V_{\text{appl}} - V_{\text{fb}}}, \tag{4}$$

where V_{appl} is the applied potential and V_{fb} the flatband potential. Within this RC description, the resistances associated with each surface state are obtained from the time constants,

$$R_{\text{fast}} = \frac{\tau_{\text{fast}}}{C_{\text{fast}}}, \qquad R_{\text{slow}} = \frac{\tau_{\text{slow}}}{C_{\text{slow}}}. \tag{5}$$

As described in the main text, we assign the fast charging process to the recombination of holes stored in monodentate coordinated surface OH groups and the slow to recombination of holes stored in bidentate coordinated surface OH groups,

$$R_{\text{fast}} \equiv R_{\text{mono}}, \quad C_{\text{fast}} \equiv C_{\text{mono}}, \quad R_{\text{slow}} \equiv R_{\text{bi}}, \quad C_{\text{slow}} \equiv C_{\text{bi}}, \tag{6}$$

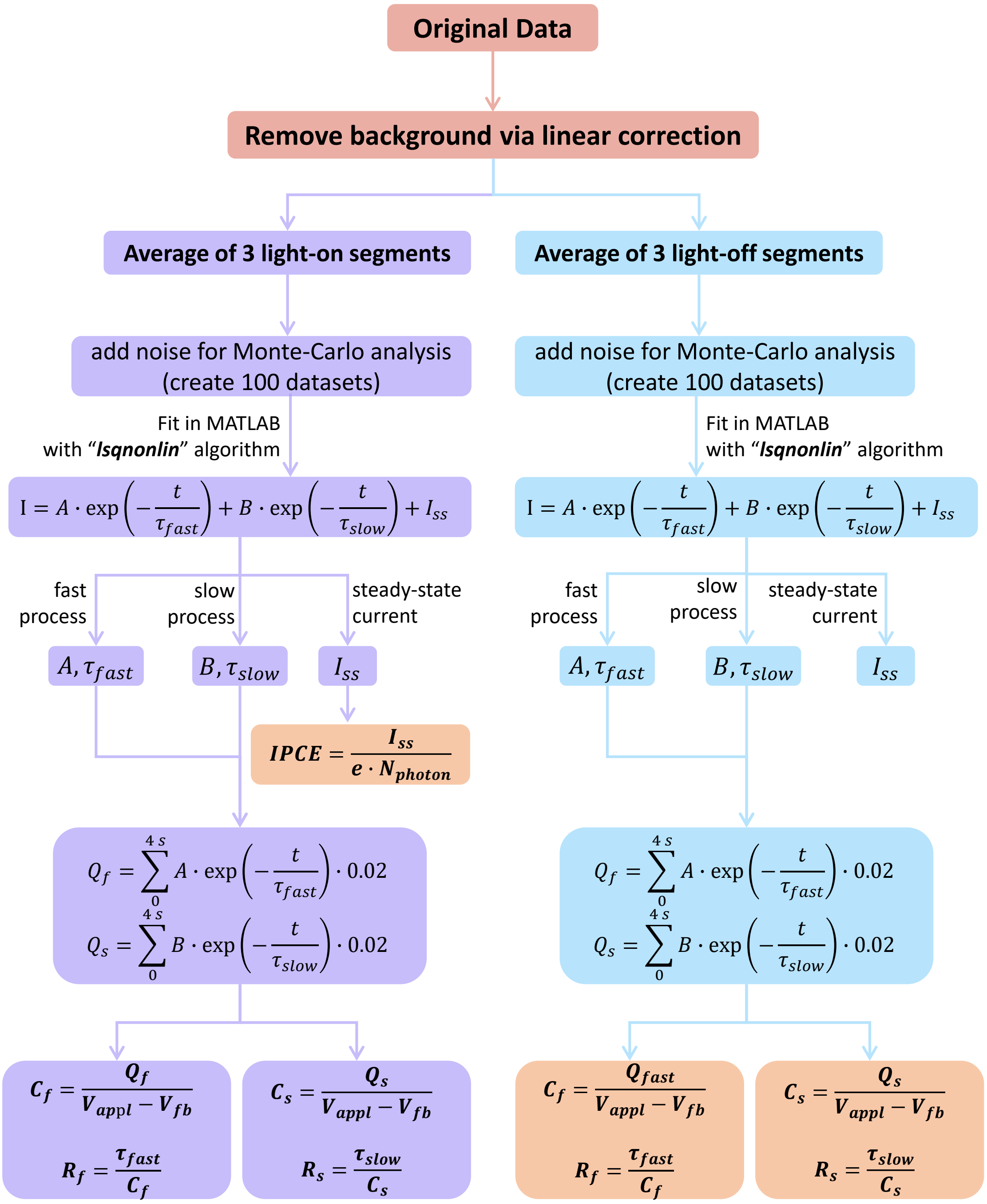

Figure S5: Flow chart of the data-processing workflow used to extract the parameters from the transient photocurrent measurements.

To quantify the uncertainty of the extracted parameters and to obtain the error bars shown in Figs. 3–5, we performed a Monte-Carlo analysis. Starting from the averaged data of 3 segments for each potential and wavelength, we generated 100 synthetic data sets by adding zero-mean Gaussian noise with standard deviation $\sigma = 0.01 \cdot \max(I_{\text{ori}})$ (1% of the maximum absolute current in the averaged transient; corresponding full width at half maximum (FWHM)is $\text{FWHM} = 2\sqrt{2 \ln 2}\sigma \approx 2.355\,\sigma \approx 0.0236 \cdot \max(I_{\text{ori}})$), where I_{ori} denotes the original dataset. Each synthetic data set was refitted with the same bi-exponential model, yielding distributions of A, B, τ_{fast} and τ_{slow}, and thus of Q_{fast}, Q_{slow}, C_{fast}, C_{slow}, R_{fast} and R_{slow}. The mean values of these distributions are reported in the figures, and their standard deviations are used as error bars.

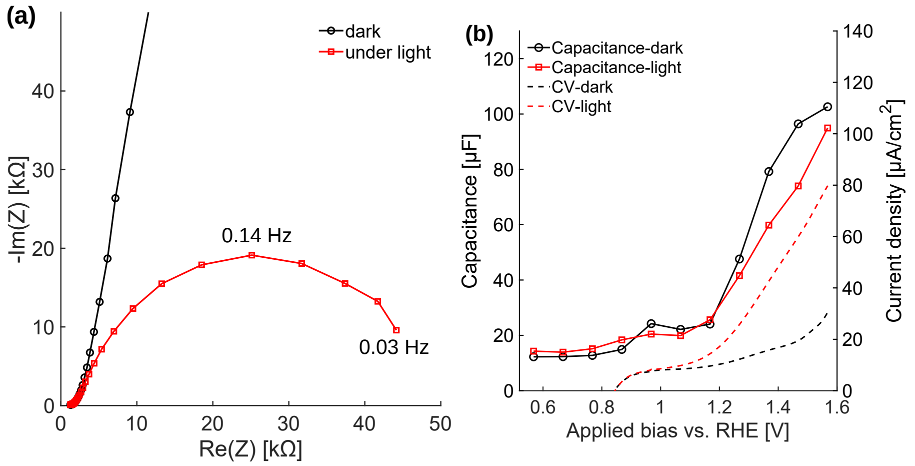

Figure S6: EIS spectra and fitted capacitance compared with CV. (a) Nyquist plot at 1.17V. Below 30 kHz, only a single semicircle is observed over all applied potentials. These data are well described by a single Randles-type element with a smoothly varying capacitance and no additional low-frequency arc that would indicate a separate, slow double-layer charging process. Given the relatively low frequency resolution of this measurement it is apparent that the two relaxation processes we observe in the time-domain can be described by a single effective RC element. (b) The extracted capacitance (dotted line) is plotted together with the CV. Near the OER onset potential, a capacitance peak appears, consistent with literature reports, and is attributed to charge accumulation. Above the OER onset, the capacitance increases with bias, consistent with an increasing contribution from reaction capacitance that is not cleanly separable in the single-element fit. The overall capacitance behavior indicates that the surface does not experience significant Fermi-level pinning with increasing bias.

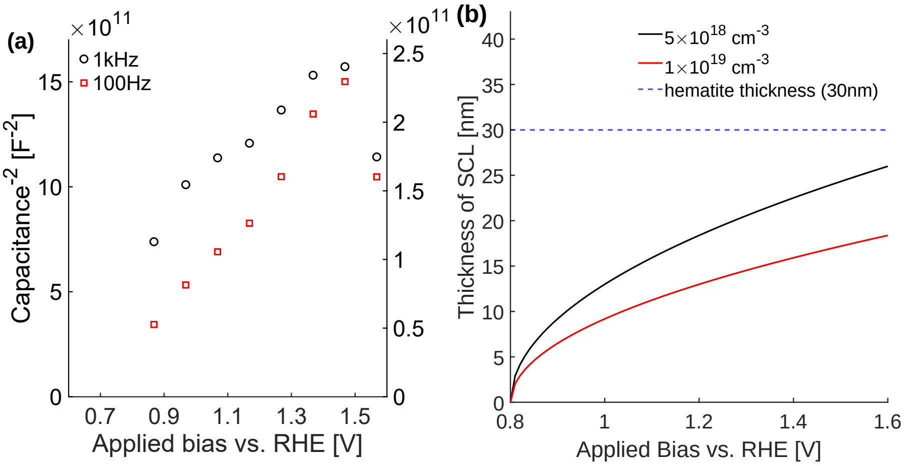

Figure S7: (a) Mott–Schottky analysis from staircase potentio-electrochemical impedance spectroscopy (SPEIS, performed at potentials ranging from 0.87 V to 1.57 V in 0.1 V increments), yielding an electron donor density on the order of $N_D \sim 10^{19}\,\mathrm{cm}^{-3}$. (b) Depletion-approximation estimate of the space-charge layer (SCL) thickness using the extracted N_D. Across the measured potential window, the SCL remains thinner than the hematite film, indicating that band bending is confined within the hematite layer (i.e. the film is not fully depleted).

To estimate the majority-carrier donor density of the hematite film we performed staircase potentio-electrochemical impedance spectroscopy (SPEIS) and extracted the space-charge capacitance as a function of applied potential. At each bias step we obtain the complex impedance $Z(\omega) = Z'(\omega) + iZ''(\omega)$. The capacitance associated with the high-frequency (space-charge) response is obtained directly from the imaginary part at a chosen frequency (where the response is predominantly capacitive). For an n-type semiconductor in depletion, the Mott–Schottky relation is[5]

$$\frac{1}{C_{\mathrm{sc}}^2} = \frac{2}{\varepsilon\varepsilon_0 e N_{\mathrm{D}} A^2}\left(V_{\mathrm{appl}} - V_{\mathrm{fb}} - \frac{k_B T}{e}\right), \tag{7}$$

where C_{sc} is the depletion (space-charge) capacitance, A is the electrode area, ε is the relative permittivity of hematite, ε_0 the vacuum permittivity, e the elementary charge, k_BT the thermal energy, V_{appl} the applied potential (vs. RHE), and V_{fb} the flat-band potential relative to RHE.

From the extracted $C_{\mathrm{sc}}(V_{appl})$ we construct the Mott–Schottky plot, i.e. $1/C_{\mathrm{sc}}^2$ versus V_{appl}. A linear fit is performed in the depletion region where the plot is approximately linear (0.87–0.97 V for 1 kHz, and 0.87–1.27 V for 100 Hz). The SPEIS-derived Mott–Schottky plots show partial nonlinearity because the extracted capacitance is not purely the space-charge capacitance. Frequency dispersion and bias-dependent contributions from reaction (chemical) capacitance, especially after the OER onset, distort the ideal linear $1/C^2$–V relation.

The donor density is obtained from the fitted slope m as

$$N_D = \frac{2}{\varepsilon\varepsilon_0 e A^2 m} \tag{8}$$

The flat-band potential is taken from the x-intercept of the linear fit,

$$V_{\text{fb}} \approx V\Big|_{1/C_{\text{sc}}^2 \to 0} - \frac{k_B T}{e}, \tag{9}$$

where the thermal term ($k_B T/e \approx 25.7$ mV at 298 K) is small compared with the experimental voltage scale.

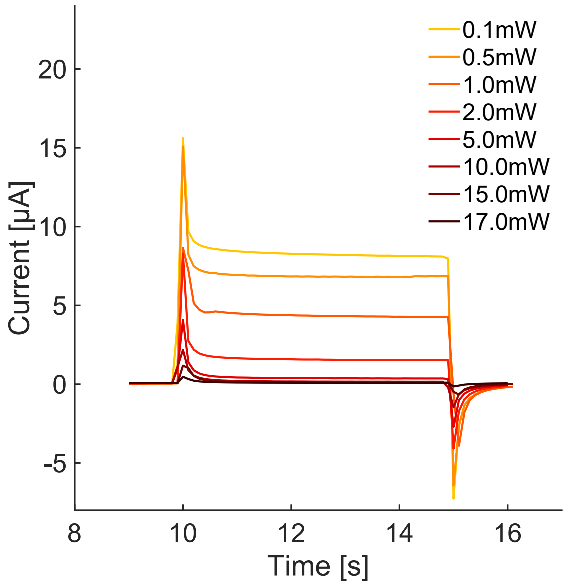

Figure S8: Photocurrent under CA conditions (1.3 V vs. RHE) as a function of illumination power. Each curve exhibits a pronounced photocurrent spike at the onset of illumination, followed by a decay to a steady-state value. The consistent transient shape across all illumination powers suggests that the photocurrent transients are not influenced by the incident photon fluence.

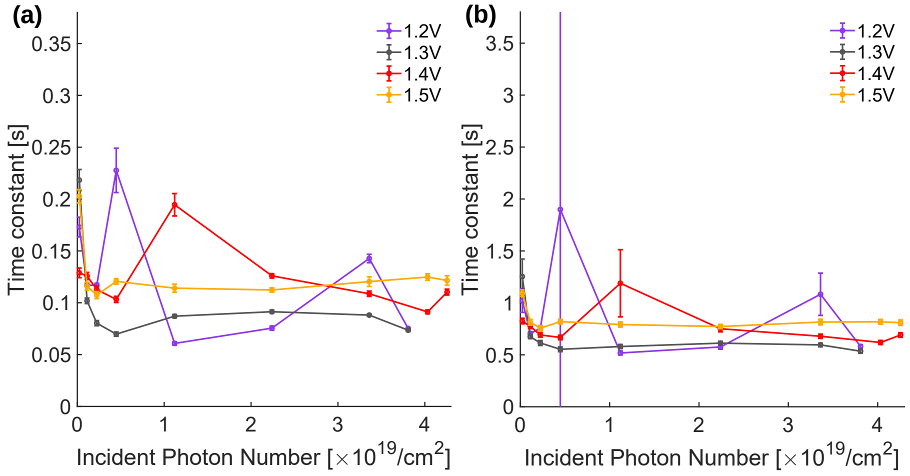

Figure S9: Fitted (a) fast and (b) slow time constants extracted from the current transients in Fig. S8 for 445 nm illumination at different incident power densities. The decay times are independent of photon flux, indicating that increasing light intensity mainly scales the transient amplitudes and the steady-state current without changing the underlying dynamics. The unusually large error bar at 1.2 V in (b) arises from the very small photocurrent under this condition, which leads to a higher relative uncertainty of the fitted time constant.

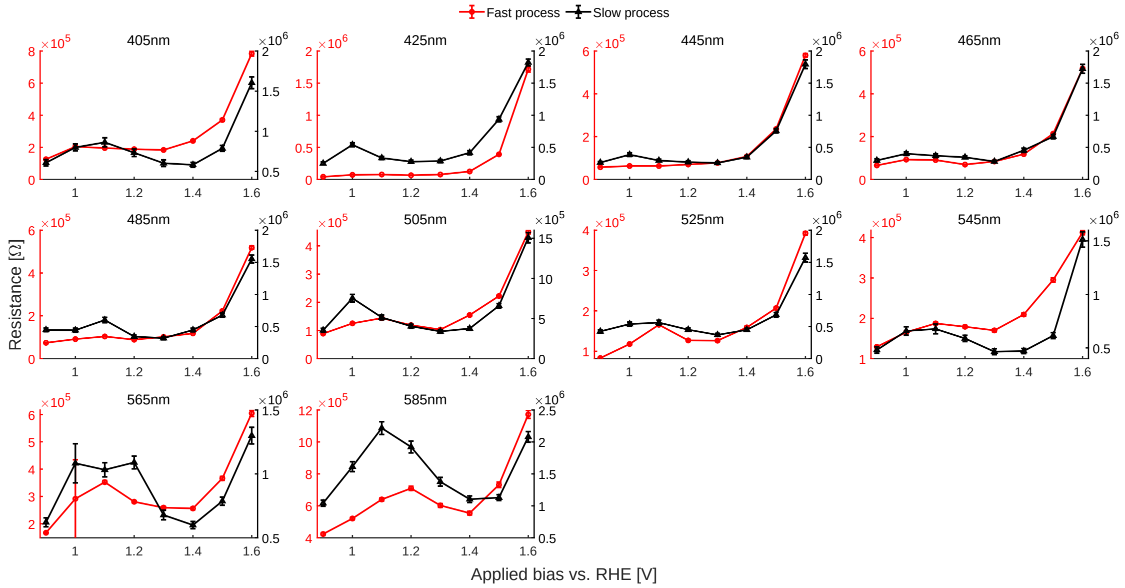

Figure S10: Resistance as a function of applied bias at different wavelengths under dark conditions.

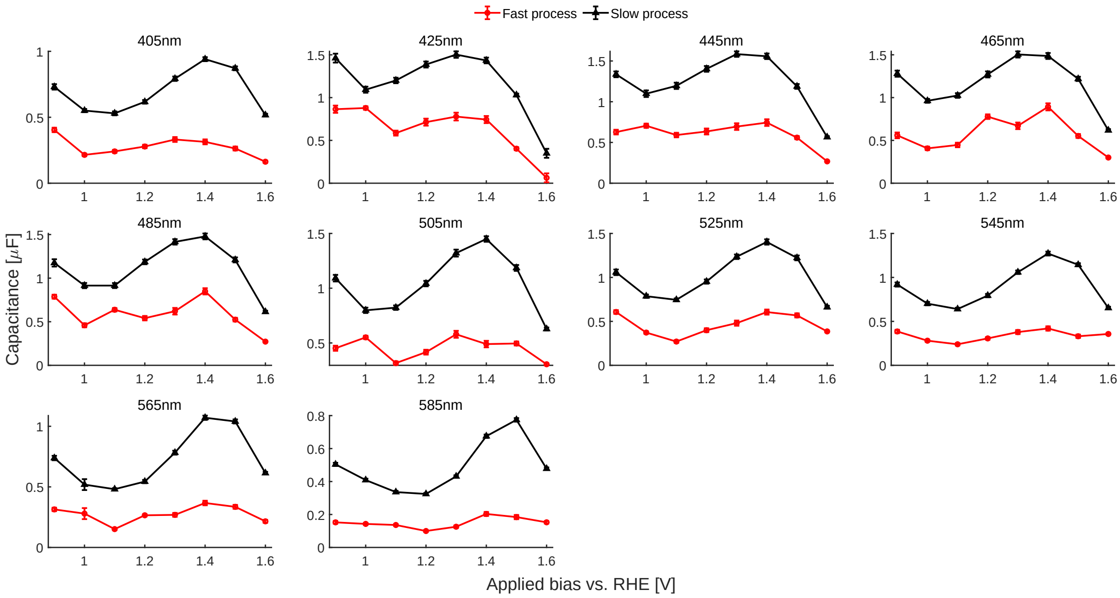

Figure S11: Capacitance as a function of applied bias at different wavelengths under dark conditions.

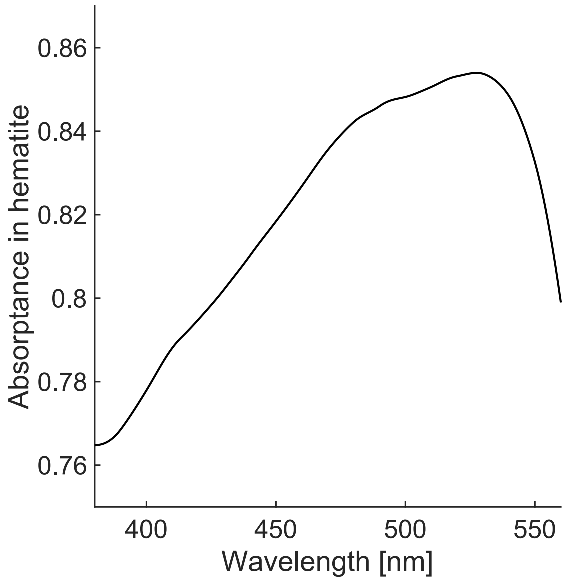

Figure S12: Theoretical hematite-layer absorptance calculated by a coherent transfer-matrix model (TMM). The wavelength-dependent absorptance $A_{\mathrm{hema}}(\lambda)$ of the 30 nm hematite layer in a semi-infinite electrolyte/hematite(30 nm)/Pt stack under normal incidence was calculated using a coherent transfer-matrix method with literature complex refractive indices $\tilde{n}(\lambda) = n(\lambda) + ik(\lambda)$ for hematite and Pt. The curve represents the fraction of incident optical power dissipated within the hematite layer after accounting for multiple reflections and interference in the thin-film stack. A reduced A_{hema} is obtained toward the short-wavelength end of the scan (including ∼405 nm) compared to the 425–445 nm region. Under the constant-photon-flux protocol used for the IPCE measurements, the rapid IPCE attenuation at 405 nm can be understood as the combined consequence of (i) the reduced hematite-layer absorptance in the electrolyte/hematite/Pt stack and (ii) the intrinsically weaker optical transition strength around 400–420 nm (often discussed as a LMCT-related "valley"), which together suppress the steady-state photocurrent I_{ss} and thus the IPCE at 405 nm.

Transfer-matrix / Fresnel thin-film optics for absorptance of hematite on Pt

Optical model and assumptions. The wavelength-dependent absorptance of a 30 nm hematite film on Pt was calculated using coherent thin-film optics at normal incidence. The optical stack is treated as a semi-infinite electrolyte / hematite film / semi-infinite Pt substrate.

Optical constants. Each medium is described by a complex refractive index $\tilde{n}(\lambda) = n(\lambda) + ik(\lambda)$. The electrolyte was approximated as non-absorbing in

the visible, with $\tilde{n}_0(\lambda) \approx n_0 = 1.33$ and $k_0 \approx 0$ (water-like refractive index)[6]. The hematite optical constants $\tilde{n}_1(\lambda)$ were taken from Optical constants by Querry[7]. The Pt optical constants $\tilde{n}_2(\lambda)$ were taken from Werner et. al[8]. All $n(\lambda), k(\lambda)$ datasets were interpolated onto a common 1 nm wavelength grid before evaluation.

Coherent reflection and internal field amplitudes. At normal incidence, the Fresnel amplitude coefficients between media j and k are

$$r_{jk} = \frac{\tilde{n}_j - \tilde{n}_k}{\tilde{n}_j + \tilde{n}_k}, \qquad t_{jk} = \frac{2\tilde{n}_j}{\tilde{n}_j + \tilde{n}_k}. \tag{10}$$

Let $\tilde{n}_0$ denote the electrolyte, $\tilde{n}_1$ hematite, and $\tilde{n}_2$ Pt. For a hematite thickness d the complex phase thickness can be defined

$$\beta(\lambda) = \frac{2\pi}{\lambda}\,\tilde{n}_1(\lambda)\,d. \tag{11}$$

The coherent reflection amplitude of the electrolyte/hematite/Pt stack is then[9;10]

$$r(\lambda) = \frac{r_{01} + r_{12}e^{2i\beta}}{1 + r_{01}r_{12}e^{2i\beta}}, \qquad R(\lambda) = |r(\lambda)|^2. \tag{12}$$

For an incident electric-field amplitude E_{inc} in the electrolyte, the forward and backward field amplitudes inside hematite at $z = 0^+$ (electrolyte/hematite interface, hematite side) are obtained from the same infinite-sum (equivalently transfer-matrix) solution:

$$E^+(0^+) = \frac{t_{01}E_{\text{inc}}}{1 + r_{01}r_{12}e^{2i\beta}}, \qquad E^-(0^+) = E^+(0^+)\,r_{12}\,e^{2i\beta}. \tag{13}$$

These expressions account for all multiple reflections between the top (electrolyte/hematite) and bottom (hematite/Pt) interfaces.

Absorptance of the hematite film from Poynting-flux balance. The electric field in hematite is written as a superposition of forward and backward waves,[9]

$$E(z) = E^+e^{ik_0\tilde{n}_1 z} + E^-e^{-ik_0\tilde{n}_1 z}, \qquad k_0 = \frac{2\pi}{\lambda}. \tag{14}$$

For non-magnetic media ($\mu = \mu_0$), the corresponding magnetic field is[9]

$$H(z) = \frac{\tilde{n}_1}{\eta_0}\left(E^+e^{ik_0\tilde{n}_1 z} - E^-e^{-ik_0\tilde{n}_1 z}\right), \tag{15}$$

and the time-averaged normal Poynting flux is[9]

$$S(z) = \frac{1}{2}\text{Re}[E(z)H^*(z)]\,. \tag{16}$$

The absorptance of the hematite film is then computed as the net flux loss across the film,

$$A_{\text{hema}}(\lambda) = \frac{S(0^+) - S(d^-)}{S_{\text{inc}}}, \qquad S_{\text{inc}} = \frac{1}{2}\frac{\text{Re}(\tilde{n}_0)}{\eta_0}|E_{\text{inc}}|^2. \tag{17}$$

Note on the incident-intensity convention. Throughout, A_{hema} is normalized to the incident Poynting flux in the electrolyte at the electrolyte/hematite interface (i.e. the intensity of the wave impinging on the hematite surface). Therefore, external coupling losses occurring above the electrolyte (*e.g.* window reflections) do not enter this definition and can be treated separately if needed.

References

[1] D. L. A. De Faria, S. Venâncio Silva and M. T. De Oliveira, *Journal of Raman Spectroscopy*, 1997, **28**, 873–878.

[2] O. Zandi and T. W. Hamann, *Physical Chemistry Chemical Physics*, 2015, **17**, 22485–22503.

[3] C.-M. Liu and J.-C. Chen, *Applied Physics Letters*, 2006, **89**, 011912.

[4] X. Luo, L. Wen, L. Zhou and Y. Yuan, *International Journal of Environmental Research and Public Health*, 2023, **20**, 4247.

[5] A. Hankin, F. E. Bedoya-Lora, J. C. Alexander, A. Regoutz and G. H. Kelsall, *Journal of Materials Chemistry A*, 2019, **7**, 26162–26176.

[6] G. M. Hale and M. R. Querry, *Applied Optics*, 1973, **12**, 555–563.

[7] M. R. Querry, *Optical Constants*, CRDC-CR-85034, 1985.

[8] W. S. M. Werner, K. Glantschnig and C. Ambrosch-Draxl, *Journal of Physical and Chemical Reference Data*, 2009, **38**, 1013–1092.

[9] H. A. Macleod and H. A. Macleod, *Thin-film optical filters*, CRC press, 2010.

[10] C. C. Katsidis and D. I. Siapkas, *Applied Optics*, 2002, **41**, 3978–3987.
